# Long-term time trends in reactivated herpes simplex infections and treatment in Sweden

**DOI:** 10.1186/s12879-022-07525-w

**Published:** 2022-06-15

**Authors:** Karin Lopatko Lindman, Judith Lockman-Lundgren, Bodil Weidung, Jan Olsson, Fredrik Elgh, Hugo Lövheim

**Affiliations:** 1 Department of Community Medicine and Rehabilitation, Geriatric Medicine, Umeå University, Umeå, Sweden; 2 Department of Public Health and Caring Sciences, Geriatric Medicine, Uppsala University, Uppsala, Sweden; 3 Department of Clinical Microbiology, Umeå University, Umeå, Sweden; 4 Wallenberg Centre for Molecular Medicine (WCMM), Umeå University, Umeå, Sweden

**Keywords:** Herpes simplex, Antiviral agents, Cohort study, Apolipoprotein E4, Ultraviolet radiation, Epidemiology, Seroprevalence

## Abstract

**Background:**

Our aim was to describe the annual prevalence of herpes simplex virus (HSV) reactivation in relation to solar ultraviolet (UV) radiation and antiviral drug use in the Swedish adult population.

**Methods:**

The study comprised 2879 anti-HSV-1 immunoglobulin (Ig) G positive subjects from five different cohorts who had donated serum from 1988 to 2010. The sera were analyzed for anti-HSV IgM using enzyme-linked immunosorbent assay. Associations between the presence of anti-HSV IgM antibodies, the apolipoprotein E ε4 allele and the serum sampling year were assessed by logistic regression. Seasonality of anti-HSV IgM was evaluated in a UV radiation model.

Data of antiviral drugs for the entire Swedish population were compiled from two different nationwide databases: the Swedish Prescribed Drug Register and the Swedish Association of the Pharmaceutical Industry.

**Results:**

Cross-sectional and longitudinal analyses indicated that the prevalence of anti-HSV IgM antibodies declined between 1988 and 2010 (odds ratio [OR] = 0.912, p < .001), while the total annual use of antiviral drugs in Sweden gradually increased from 1984 to 2017. Higher UV radiation was associated with higher prevalence of anti-HSV IgM antibodies (OR = 1.071, p = .043).

**Conclusion:**

The declining time trend of HSV reactivation in a Swedish cohort coincides with a steady increase of antiviral drug use in the Swedish general population.

**Supplementary Information:**

The online version contains supplementary material available at 10.1186/s12879-022-07525-w.

## Introduction

Several studies have indicated a potential link between recurrent herpes simplex virus 1 (HSV-1) infection and the risk of Alzheimer’s disease (AD) [1–3]. Also, recent findings suggest that antiviral treatment might offer some protection against the incidence of major neurocognitive disorders [4–6].

Herpesviruses are ubiquitous and an estimated 80% of the Swedish adult population is seropositive for HSV-1, 13% for HSV-2 and 98% for varicella zoster virus (VZV) [7]. Recurrent HSV and VZV infections are often treated with antiviral drugs, such as acyclovir and its prodrug valacyclovir that targets actively replicating herpesviruses, to reduce the duration of symptoms and viral shedding [8, 9].

Previous studies have reported a declining incidence of AD and other neurocognitive disorders over the last decade [10–12]. Before, the age-adjusted incidence had been relatively stable during the last 50 years [13]. A potential contributing factor for the declining incidence of AD could be the introduction of antiviral drugs against herpesviruses, at least in the subset of the population with HSV infection. This hypothesis is supported by the results from recent register-based studies, where use of antivirals was associated with a lower risk of major neurocognitive disorders [4–6].

Previous reports of HSV-1 epidemiology have primarily focused on the seroprevalence of anti-HSV IgG and self-reported HSV reactivations, rather than time trends in reactivated infections [14–16]. While serological evidence of anti-HSV-1 IgG indicates carriage of the pathogen, anti-HSV IgM might reflect more recent viral activity, like primary or recurrent infection. In addition, only a few studies have specifically investigated the consumption rates of antiviral drugs in the general population [17]. Here, we set out to evaluate the time trend in the total use of antivirals using nationwide registries for the total population of Sweden. Also, longitudinal data for HSV-1 reactivation, measured as presence of anti-HSV IgM, was assessed for a population-based cohort in Sweden.

## Method

### Antiviral drugs

The annual prevalence of antiviral use between 1984 and 2017 were extracted from two nation-wide databases—the Swedish Prescribed Drug Register (SPDR) and the Swedish Association of the Pharmaceutical Industry (LIF). Anatomical Therapeutic Chemical (ATC) codes J05AB (J05A direct- acting antivirals: subgroup J05AB nucleosides and nucleotides excl. reverse transcriptase inhibitors) were used to identify drugs in SPDR and LIF. SPDR has had full coverage since 2006 and includes all prescription drugs dispensed by Swedish pharmacies [18]. In LIF, data were available on all doses delivered to Swedish pharmacies between 1984 and 2007.

**Fig. 1:**
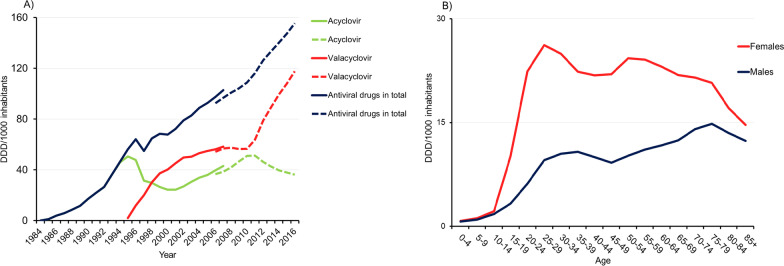
The use of antivirals in Sweden. The solid line in the diagram represents drug data collected from the LIF (1984 to 2007) and the dashed line represents SPDR data (2006 to 2017). **A** The use of antivirals in DDD/1000 inhabitants per year from 1984–2016 in Sweden, collected from two databases, the Swedish Association of the Pharmaceutical Industry (1984–2007; solid line) and the Swedish Prescribed Drug Register (2006–2016; dashed line). **B** The number of subjects with antivirals/1000 inhabitants stratified by sex and age group in 2017 in Sweden

The Defined Daily Dose (DDD) per year was calculated according to the guidelines of the World Health Organization Collaborating Centre for Drug Statistics Methodology. DDDs per 1000 inhabitants per year were calculated by dividing DDDs by the size of the Swedish population on 31 December each year, based on data obtained from Statistics Sweden and then multiplying the sum by 1000. SPDR also includes the number of treated subjects per 1000 inhabitants per year stratified by age group and sex (Fig. 1B, showing data for 2017 and see Additional file 1 for population statistics).

### The Betula study

The Betula study is a prospective population-based cohort [19, 20]. The study comprises five cohorts (samples [S] 1–5) enrolled at different time points at intervals of 5 years between 1988–1990 and 2008–2010 (time points [T] 1–5). Cross-sectional data from the first assessments of cohorts S1–5 and longitudinal data from cohorts S1 and S3 were available in the present study. S1 and S3 were monitored every 5 years until 2008–2010. The methods of selection and serological testing is described elsewhere [19–21]. In our study, only anti-HSV-1 IgG seropositive subjects were included.

### Serum analyses

In short, a commercial enzyme-linked immunosorbent assay (ELISA) kit was used for the detection of anti-HSV1 IgG (Cat # EL0910G) (HerpeSelect1, FOCUS Diagnostics) and an in-house ELISA was used for detection of anti-HSV IgM as previously described [1].

The subjects’ first serum sample was used to determine anti-HSV IgG status. If positive, this sample was further analyzed for specific anti-HSV-1 IgG, indicating carriage of HSV-1. Anti-HSV-1 IgG-positive samples were then analyzed for the presence of anti-HSV IgM, a marker of recent viral activity. The IgM ELISAs did not distinguish anti-HSV-1 from anti-HSV-2 IgM, and a positive sample may originate from reactivated HSV-1, reactivated HSV-2 or a primary infection with HSV-2.

Apolipoprotein E (APOE) genotype was determined with a PCR-based assay as previously described [22]. For samples where DNA was unavailable, an APOEε4 specific ELISA (Cat # K4699-100) (BioVision Inc., CA, USA) was employed for phenotypic assessment.

### UV data

Data of daily ultraviolet(UV) radiation in Umeå, Sweden between 1991 and 1996 were publicly available and extracted from The Swedish Meteorological and Hydrological Institute (SMHI), registered according to previous publications [23, 24]. The UV radiation was measured in minimum erythema dose (MED) per day where one MED is equal to the CIE-weighted irradiation of 210 Jm^−2^. SMHI reports the mean daily MED value. The mean MED over five consecutive years was computed for each day of the year. Subjects were then assigned the mean MED matching their sampling day of the year. We estimated a non-linear regression curve since the plotted MED raw data varied greatly between summer and winter. A multiplicative function of three sine waves provided a visually adequate fit to the data points.

### Statistics

The use of antivirals (total J05AB, as well as separately for the most common substances, acyclovir and valacyclovir) was calculated in the entire Swedish population every year from 1984 to 2017 to evaluate temporal trends. Herein, the term of “antiviral drugs in total” denotes all antiviral drugs with ATC codes J05AB. The use of antivirals was also stratified by age and sex in 2017, to describe the sex- and age-specific treatment patterns.

A multivariable logistic regression model was fitted to anti-HSV IgM with sex, age, carriage of APOEε4 (presence of one or two ε4 alleles), sample year and UV radiation as covariates in the cross-sectional sample. We assured that the assumptions were met before performing the logistic regression analysis using the Box-Tidwell test and by plotting the independent variables against the prevalence of anti-HSV IgM, to test linearity between the continuous independent variables and the log odds. Variance inflation factor (VIF) values were computed for each independent variable, and VIF > 2.5 was considered a threshold for multicollinearity.

The prevalence of anti-HSV IgM antibodies was examined cross-sectionally. For cohorts S1 and S3 the prevalence of anti-HSV IgM was also assessed longitudinally. In the cross-sectional analyses, the subject’s first available sample was used from time points T1–T4. In the longitudinal models, two cohorts (S1 and S3) were monitored from T1 (S1) and T2 (S3), respectively, until T5. Subgroup analyses were performed by sex and carriage of APOEε4.

The prevalence of anti-HSV IgM for samples taken each day of the year was also plotted against solar UV radiation levels to visualize any seasonal variations in HSV infection. A time lag of 19 days between the exposure (MED function) and the outcome (simple sine function fitted to prevalence of IgM antibodies at each day of the year) was observed. The nineteen-day lag was subsequently used to account for the potential delay in IgM response after sun exposure when UV radiation _(t=-19)_, was added to the logistic regression model of anti-HSV IgM positivity. To compare the odds of anti-HSV IgM seropositive samples between summer and winter, the odds ratio (OR) of anti-HSV IgM positivity with UV radiation _(t=-19)_ was raised to the power of the mean MED difference between summer months (June–August) and winter months (December–February).

Statistical analyses were performed using SPSS Statistics Version 25 (IBM Corporation, Armonk, NY) and R version 4.1.3. Diagrams were constructed in Microsoft Excel (Microsoft Corporation, Redmond, WA). A p value < 0.05 was interpreted as significant.

### Ethical approval

The study was performed in accordance with the Declaration of Helsinki and was approved by the Regional Ethical Review Board in Umeå, Sweden (2010-229-31 M). All participants of the Betula study provided written informed consent for research on stored blood samples and access to medical records.

## Results

### Antiviral drugs

Figure 1A shows the annual prevalence of antiviral use between 1984 and 2016. Since the introduction of acyclovir in 1984, there has been an almost linear increase in the overall use of antivirals (Fig. 1A). Acyclovir shows a pattern with two peaks, one in 1995 and the other in 2011. In contrast, the use of valacyclovir increased sharply after its introduction in 1995 and then reached a plateau. However, after 2010, the use of valacyclovir increased sharply again (Fig. 1A).

Differences were observed in the use of antiviral drugs between males and females. In 2017, a higher proportion of females seemed to be using antiviral drugs compared to males, across all age groups (Fig. 1B).

### Anti-HSV IgM

In the present study, 2879 subjects with anti-HSV-1 IgG antibodies were included from five different cohorts. 96 of these subjects (3.3%) tested positive for anti-HSV IgM at the first individual serum sampling. With the exception of the summer months, the collection of blood samples was spread evenly throughout the year (30.7% collected during winter, December–February, 30.5% collected during spring, March–May, 6.5% collected during summer, June–August, and 32.3% collected during autumn, September–November). The prevalence of anti-HSV IgM at different sampling intervals and the descriptive statistics are presented in Table 1 and in a flow chart, see Additional file 2.

**Table 1:** Descriptive statistics of cohorts S1 to S5

Cohorts	S1	S2	S3	S4	S5
n	764	774	638	278	425
Age range, y*	35–91	35–81	59–90	60–91	35–95
Age, y, mean ± SD^a^	61.7 ± 14.1	59.9 ± 14.0	69.8 ± 9.0	74.1 ± 9.2	62.9 ± 16.0
Female, n (%)	411 (53.8)	436 (56.3)	376 (58.9)	139 (50.0)	218 (51.3)
APOEε4 carriers, n (%)	227 (29.7%)	226 (29.2%)	183 (28.7%)	72 (25.9%)	89 (20.9%)
Sampling year, n Anti-HSV IgM positive samples/n (%)					
T1: 1988–1990	30/590 (5.1)				
T2: 1993–1995	12/454 (2.6)	30/774 (3.9)	17/492 (3.5)		
T3: 1998–2000	27/381 (7.1)		29/471 (6.2)	9/278 (3.2)	
T4: 2003–2005	3/339 (0.9)		1/386 (0.3)		3/425 (0.7)
T5: 2008–2010	1/246 (0.4)		1/222 (0.5)		
First sample, n anti-HSV IgM positive samples/n (%)	35/764 (4.6)	30/774 (3.9)	19/638 (3.0)	9/278 (3.2)	3/425 (0.7)

n: number; y: years; SD: standard deviation; APOEε4: apolipoprotein E allele 4; IgM” Immunoglobulin M

^a^On the date of the first included serum sample

**Fig. 2:**
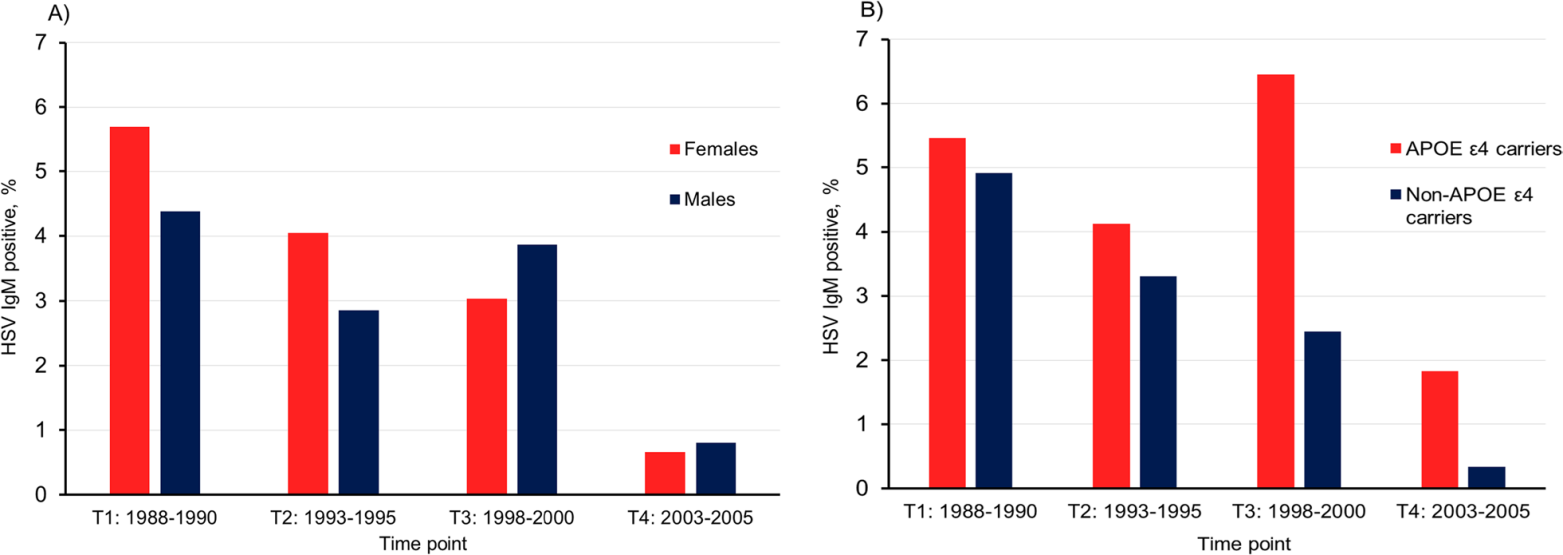
Anti-HSV IgM positivity in the Betula cohort. **A** Cross-sectional comparisons of the prevalence of anti-HSV IgM at first sampling between males and females at different time points. **B** Cross-sectional comparisons of the prevalence of anti-HSV IgM at first sampling between APOEε4 carriers and non-APO*E*ε4 carriers

In the cross-sectional analyses, the highest proportion of anti-HSV IgM positive samples at inclusion was observed at T1 (Table 1). For subsequent time points, the proportion of anti-HSV IgM positive samples were lower, and in particular at T4 (Table 1). The prevalence of anti-HSV IgM positive status among males and females are demonstrated in Fig. 2A. Although not statistically significant, we found a higher prevalence of anti-HSV IgM among APOEε4 carriers compared to non-carriers, at all different time points (Fig. 2B).

**Fig. 3:**
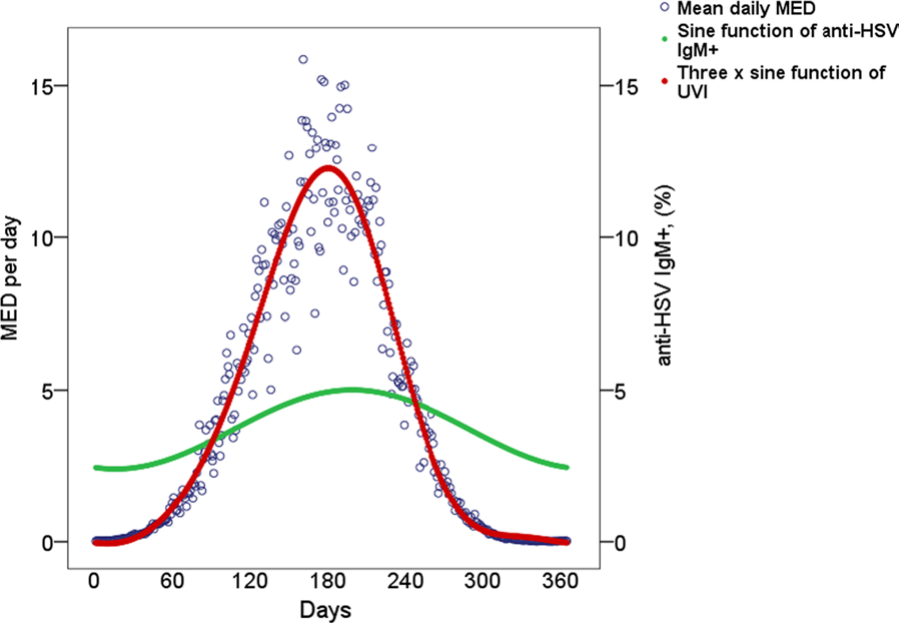
Anti-HSV IgM positivity in the Betula cohort. The prevalence of anti-HSV IgM at first sampling per sampling day of the year and daily mean UV levels in Umeå, Sweden. The prevalence of anti-HSV IgM antibodies for each day of the year in the Betula cohort, is plotted against daily UV levels (not individual doses) in Umeå. The blood samples in the Betula cohort were drawn between 1988 and 2005, while the mean UV value for each day was calculated as the mean over five consecutive years between 1991 and 1996. The x axis represents January 1 through December 31

Later sample year was associated with a lower prevalence of anti-HSV IgM antibodies (OR = 0.912 per year, p < 0.001, Table 2), while UV radiation _(t=-19)_ was associated with higher prevalence of anti-HSV IgM antibodies (OR = 1.071 per MED, p = 0.043; Table 2). The mean MED difference between summer and winter months was 9.967 which is equal to 2093.07 Jm^−2^. The odds for anti-HSV IgM positivity in summer were approximately two times higher than in winter (OR = 1.994 per mean MED difference). Figure 3 illustrates the relationship between UV radiation (measured in MED) and the prevalence of anti-HSV IgM over 1 year, where the peak prevalence of anti-HSV IgM seems to follow the UV radiation with a delay of nineteen days.

**Table 2:** Logistic regression of anti-HSV IgM with sex, age, APOEε4, sampling year and UV radiation

	Odds ratio	95% Confidence interval	*P*-value
Sex (reference: female)	0.801	0.528–1.218	.300
Age	1.009	0.993–1.024	.271
Carriage of APOEε4	1.466	0.956–2.247	.079
Sampling year	0.912	0.868–0.960	< .001
UV radiation_(t=-19),_	1.071	1.002–1.145	.043

## Discussion

Here, we investigated two possibly related time trends in two different datasets: the total use of antiviral drugs targeting herpesviruses since their introduction in 1984 until 2017 and the prevalence of anti-HSV IgM in a Swedish cohort sampled between 1988 and 2010. The overall use of antivirals in Sweden has gradually increased (Fig. 1A). In the studied cohort, the prevalence of reactivated HSV infection showed a declining trend with later sampling year being associated with lower prevalence of anti-HSV IgM (Table 2).

Our results of an overall higher antiviral drug use are in accordance with previous reports, indicating increasing antiviral treatment rates against herpes zoster in Canada and higher antiviral use among pregnant women in the United States [17, 25]. A reduction of acyclovir prescriptions after an initial peak in 1995, which was observed in the present study, could be explained by the introduction of valacyclovir in 1995. A second peak in acyclovir prescriptions occurred in parallel with the expiry of the patent for acyclovir and subsequent price cuts in 1997. Finally, a subsequent decline in acyclovir prescriptions occurred when the patent for valacyclovir expired in 2009 and the price of valacyclovir decreased. The reason for the two partially overlapping data series in Fig. 1 is because we extracted drug data from two different sources, in which LIF has information about doses delivered to pharmacies (1984 to 2007) and SPDR includes all dispensed drugs (2006 to 2016). However, data on the percentage of dispensed doses among the doses delivered to pharmacies were not available in LIF. This explains the slightly higher prevalence of antivirals in the overlapping years of 2006 and 2007 in Fig. 1A.

The decline in prevalence of anti-HSV IgM among individuals with HSV-1 in our material was demonstrated both between and within the sub-cohorts. These findings may imply an improvement in the immunological control of herpes infections. Whether, or to what extent, the increasing use of antivirals in Sweden may have contributed to this decline cannot be estimated from the present study. Notably, previous clinical trials have not detected any effect on recurrence rates with episodic acyclovir after discontinuation [8, 9].

Our results could be interpreted in the light of previous findings indicating that HSV-1 reactivation, measured as the presence of anti-HSV IgM antibodies, is associated with increased risk of subsequent AD [1, 3], while antiviral treatment might reduce this risk [4–6]. Interestingly, the incidence of major neurocognitive disorders in Sweden appears to have declined from 2010 and onwards [10]. However, whether the growing use of antiviral drugs in Sweden could have impacted the incidence of AD cannot be established from the observation of these parallel trends.

Although not significant, we also observed a tendency for APOEε4 carriers to have an increased risk of anti-HSV IgM seropositivity (Table 2 and Fig. 2B), in line with other studies indicating a link between HSV-1 and the APOEε4 allele [21, 26–28].

The distribution of anti-HSV IgM seropositive samples showed a seasonal variation and seemed to relate with the changes in UV radiation. Exposure to UV radiation is a known trigger of HSV reactivation [29] and higher UV index was previously found to associate with recurrence of ocular HSV under certain conditions [30]. Previous studies of UV radiation and HSV reactivations have mainly examined high risk groups with prior history of recurrent infections or have had small population samples. Our cohort consisting of a large, unselected sample could therefore provide additional support to the relationship between UV exposure and HSV reactivations in the general population. However, other causes of HSV reactivation during summer have to be considered.

The strengths of our investigation are that we used nationwide registers that reflect drug prescriptions in the entire Swedish population. Conversely, the primary strengths of the Betula cohort study relate to the large population-based sample and long follow-up period. A significant limitation is the small number of individuals positive for anti-HSV IgM antibodies and that individual drug data were not available for the cohort study population. Importantly, the IgM ELISAs did not differentiate HSV-1 from HSV-2, and a positive sample might indicate reactivated HSV-1 or HSV-2 infection, or primary infection with HSV-1 or HSV-2. Also, the collection of blood specimens was not entirely evenly distributed, with a lower number of blood samples being drawn during summer, however this should not impact the regression fitting to the sinusoidal curve. It should be noted that we did not estimate individual UV dose nor did we use measurements for UV exposure time. The figures for UV levels in this study were used as a proxy for seasonality, the rational being that it allows for further comparisons between years. However, the limited sample size in the current study only allows for estimates of years average. Likewise, we did not have access to individual antiviral data for the participants of the Betula cohort, but evaluated the total antiviral use in the entire Swedish population by registry data. Hence, our results are descriptive of the trends in two diverse measures in different samples but do not prove any causal link or direct associations, which must be taken into account when interpreting the results.

### Conclusion

Antiviral drugs have become increasingly common since acyclovir was introduced in 1984. Since 2000, there has been a marked decline in the prevalence of recent HSV infection among HSV-1 seropositive individuals, indicating improved control of herpesvirus infections in the Swedish adult population. We also noted a seasonal variation where the distribution of anti-HSV IgM seropositive samples seemed to relate with changes in UV radiation.

## Supplementary Information

**Additional file 1. **Sweden population statistics.

**Additional file 2.** Flow chart of the Betula cohort and anti-HSV IgM seropositivity in the different samples

## Data Availability

The Betula dataset used in the current study is available from the corresponding author on reasonable request. The dataset of drug prescriptions was extracted from the open database of the National Board of Health and Welfare and the Swedish Association of the Pharmaceutical Industry.

## Abbreviations

HSV: Herpes simplex virus
AD: Alzheimer’s disease
VZV: Varicella zoster virus
Ig: Immunoglobulin
SPDR: The Swedish Prescribed Drug Register
LIF: The Swedish Association of the Pharmaceutical Industry
ATC: Anatomical Therapeutic Chemical
DDD: The Defined Daily Dose
ELISA: Enzyme-linked immunosorbent assay
APOE: Apolipoprotein E
UV: Ultraviolet
SMHI: The Swedish Meteorological and Hydrological Institute
MED: Minimum erythema dose
OR: Odds ratio
