# Supplementary material for: Long-term time trends in reactivated herpes simplex infections and treatment in Sweden
Source: BMC Infect Dis. 2022 Jun 15;22:547. doi: 10.1186/s12879-022-07525-w (PMC9199307; doi:10.1186/s12879-022-07525-w)
Supplement: Supplementary file 1 — Additional file 1. Sweden population statistics. [file 12879_2022_7525_MOESM1_ESM.docx]

Sweden population statistics

**Year**  **Population on December 31th, n**

1984 8,342,621

1985 8,358,139

1986 8,381,515

1987 8,414,083

1988 8,458,888

1989 8,527,036

1990 8,590,630

1991 8,644,119

1992 8,692,013

1993 8,745,109

1994 8,816,381

1995 8,837,496

1996 8,844,499

1997 8,847,625

1998 8,854,322

1999 8,861,426

2000 8,882,792

2001 8,909,128

2002 8,940,788

2003 8,975,670

2004 9,011,392

2005 9,047,752

2006 9,113,257

2007 9,182,927

2008 9,256,347

2009 9,340,682

2010 9,415,570

2011 9,482,855

2012 9,555,893

2013 9,644,864

2014 9,747,355

2015 9,851,017

2016 9,995,153

2017 10,120,242
