# Supplementary material for: Long-term time trends in reactivated herpes simplex infections and treatment in Sweden
Source: BMC Infect Dis. 2022 Jun 15;22:547. doi: 10.1186/s12879-022-07525-w (PMC9199307; doi:10.1186/s12879-022-07525-w)
Supplement: Supplementary file 2 — Additional file 2. Flow chart of the Betula cohort and anti-HSV IgM seropositivity in the different samples [file 12879_2022_7525_MOESM2_ESM.docx]

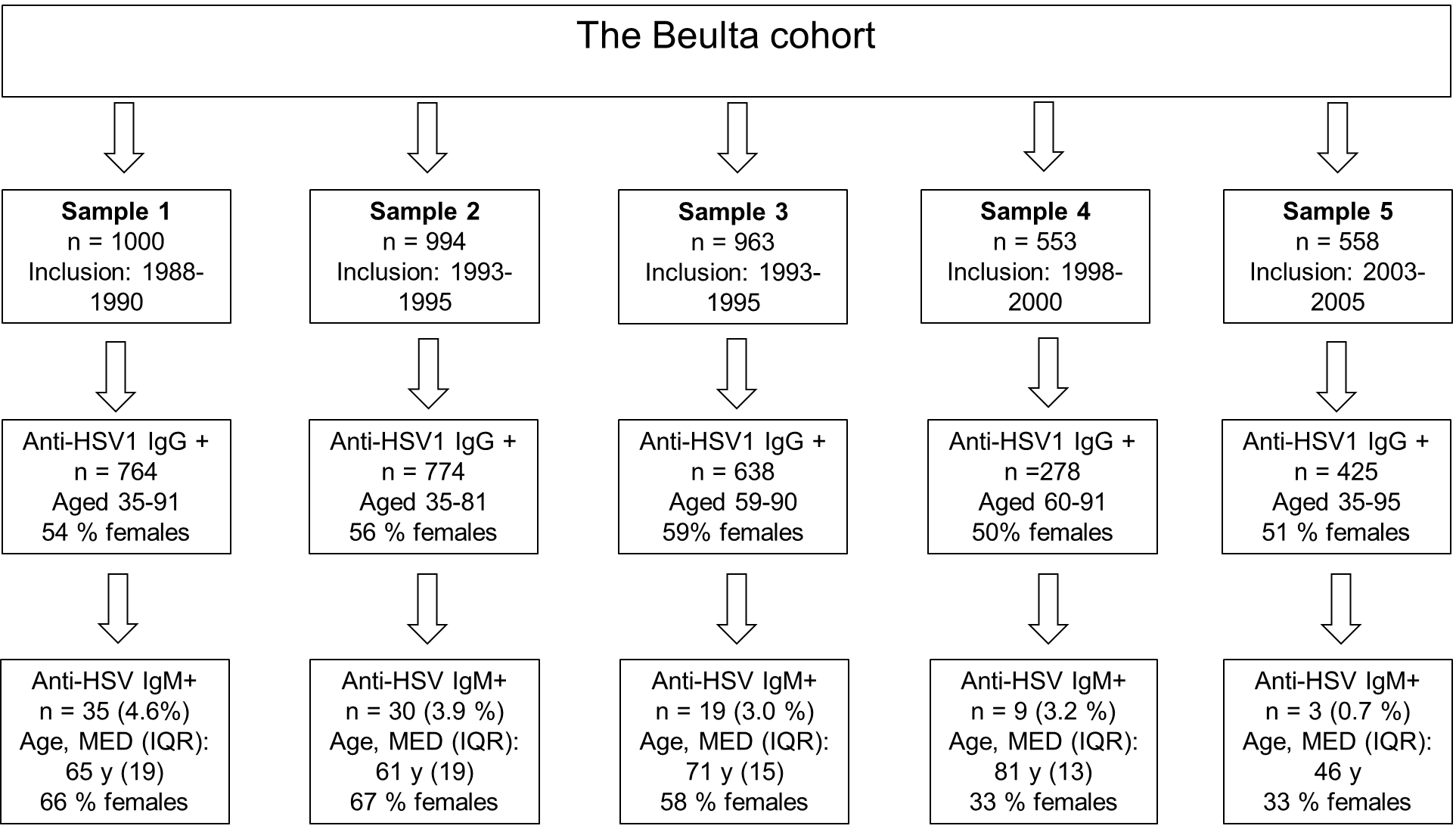
Flow chart of the Betula cohort and anti-HSV IgM seropositivity in the different samples.

MED: median
IQR: interquartile range (IQR)
